# What about the fundamentals of nursing—its interventions and its continuity among older people in need of home- or facility-based care: a scoping review

**DOI:** 10.1186/s12912-023-01675-1

**Published:** 2024-01-22

**Authors:** O. M. Nordaunet, E. R. Gjevjon, C. Olsson, H. Aagaard, G. Borglin

**Affiliations:** 1 Department of Bachelor Education (Nursing), Lovisenberg Diaconal University College, Lovisenberggata 15B, NO-0456 Oslo, Norway; 2 Institute of Health Sciences, Department of Nursing, Karlstad University, Universitetsgatan 2, 651 88 Karlstad, Sweden; 3 UiT The Arctic University of Norway, Havnegata 5, 9404 Harstad, Norway

**Keywords:** Continuity of care, Interventions, Literature review, Long-term care, Nurse, Registered nurse

## Abstract

**Aim:**

This scoping review investigated and descriptively summarised previous research about fundamental nursing, its focus (what care needs are described, how is it described and by whom is it described), continuity of care (is it described in relation to fundamental nursing) and possible nursing interventions or activities targeting older people’s fundamentals of care needs in home- or facility-based care.

**Methods:**

This scoping review was carried out following the steps of Arksey and O’Malley’s methodology and PRISMA-ScR reporting guidelines. Searches were conducted in PubMed via NIH, CINAHL via EBSCO and PsycInfo via ProQuest for the time period between January 2002 and May 2023.

**Results:**

Forty-two studies were included where the majority had been conducted in a facility-based care context. Nutrition—or rather nutritional care activities targeting eating and drinking—was the most frequently described fundamental care needs addressed. After this came personal care such as cleansing, dressing, oral care, skin, and foot care. Few studies addressed more than one fundamental care need at the time. The nursing staff described fundamental nursing as complex, comprehensive, and demanding. Older people and relatives described a gap between the fundamental nursing provided and their perceived need for support. Less attention was given to older peoples relational and psychosocial needs. Identified nursing interventions mainly targeted physical care needs. Our findings also implied that interventions focusing on fundamental nursing were described as feasible in practice with favourable or moderate results, while long-term effects were difficult to detect. No studies were identified focusing on fundamental nursing in relation to outcomes such as continuity of care.

**Conclusion:**

Fundamental nursing was mainly described in relation to physical care needs, which were essentially conducted within facility-based care contexts. Interventions and activities primarily focused on one fundamental need at the time, mainly within the physical domain. No nursing interventions were identified focusing on relational and psychosocial needs where continuity of care can be viewed as a relevant outcome. Such limited focus are especially concerning as research has highlighted the importance of that older people with complex care needs can benefit from a holistic and person-centred approach i.e. fundamental nursing.

**Trial registration:**

Open Science Framework https://doi.org/10.17605/OSF.IO/XJ39E

Protocol: http://dx.doi.org/10.1136/bmjopen-2022-069798

**Supplementary Information:**

The online version contains supplementary material available at 10.1186/s12912-023-01675-1.

## Background

The core of nursing is care [1]. Care focusing on relational, psychosocial, and physical needs such as mobility, hygiene, nutrition, and elimination is well-known by nurses as the fundamentals of care. Henderson already recognised fundamental nursing in relation to the fundamentals of care as ‘assisting people to do the things they would normally do for themselves if only they were able’ (p. 149) [2]. Hence, fundamental nursing addresses patients’ comprehensive fundamentals of care needs and is mainly portrayed as both complex and challenging care rather than as common sense [3] or basic care [4].

Recently, fundamental nursing and the fundamental care needs have attracted a lot of interest in research in nursing [4–10]. This renewed attention is likely the result of several important organisational and societal changes within the Nordic countries and Europe. We know that the reconstruction of healthcare services, such as the downsizing of specialist care (hospital care) and increase in home- or facility-based community care, has coincided with unprecedented demographical challenges [11]. European statistics have shown that the potential number of older people in home- or facility-based care is estimated to increase from about 31 million (2019) to more than 38 million by 2050 [12]. In Norway, the figures indicate that home-based nursing has increased the quickest out of all healthcare services [13]. Paralleled with this, facility-based care (here nursing homes) has, at least in the Nordic countries, gradually become a care service only for those older people with severe cognitive or physical impairment. The notion is that older people with functional disabilities should be given services at the lowest level of efficient care to remain at home as long as possible [14]. Research indicates that most of these older people are likely to value their independence, and preferer to remain in a familiar environment where they feel like they belong [15, 16]. However, many of these older people are and will be living with multimorbidity’s, which can be described as people with two or more medical diagnoses and complex care needs [17, 18] and, hence, requiring fundamental nursing over time.

It is well-known that nurses’ ability to provide care in a coordinated and meaningful way is being challenged by underfinanced, fragmented and task-oriented healthcare services [19–21]. These challenges affect both their working conditions, workloads and quality of their nursing actions while also reducing the ability to perform person-centred care [22], which impacts the continuity and quality of care. When resources are low, fundamental care needs are frequently overlooked [23]. The reasons for this vary, from understaffed wards to a devaluation of the fundamentals of care [6]. Fundamental nursing focusing on older persons’ needs and preferences consistently over time in a safe, timely, effective, efficient, equitable and person-centred manner promotes continuity and quality of care [4, 24]. However, lack of continuity of care has been found to increase hospitalisation [25, 26], mortality [27] and healthcare costs [28]. Older people have reported to complain about the involvement of different professionals in their care, lack of coordination and continuity of care [29].

Bentzen et al. [30] stated that high work pressure leads to having to choose which fundamental need to address and which to down-prioritise, sometimes at the cost of patient safety. Research shows that nurses are valued by older persons to ensure optimal and safe care [7, 8, 24], raising the argument that available, competent and skilful nurses’ matter. Currently, research into nursing regarding the fundamentals of care has mainly focused on the secondary care context [9]. Research conducted in the latter area highlights that care needs, such as oral care, hygiene and mobilisation, are overlooked or down-prioritised [31]. In contradiction, Mandal et al. [32] have revealed that pain management, medication administration and technology-oriented tasks are rarely overlooked or down-prioritised by nurses. Overall, it appears as if, in the secondary care context, the fundamentals of care might be undervalued and perceived by nursing staff as rudimental [7, 33], and of little or no value for them to engage in [8]. How transferable this is to home- or facility-based care has not yet been well described. Thus, investigating and descriptively summarising which type of fundamentals of care and what sort of interventions or activities nurses engage in related to older people in home- and facility-based care is vital to ensure care reflecting both quality and continuity in this setting. Ample research [34–36] has highlighted that older people with complex care needs would benefit from care delivered within a holistic and person-centred approach where particularly important outcomes of care, such as safety, dignity and communication, have a natural position [37, 38]. Then again, whether these latter views are shared by the older people and their significant others regarding their fundamentals of care needs is, to date, little explored within the home- or facility-based context. The same is true for the importance of continuity of care regarding caring for their needs. Pentecost et al. [10] implied that the importance of improving patients’ experiences in relation to the fundamentals of care while also promoting a consistent nursing practice and increasing attention to how nurses and patients can work together to meet patients’ individual care needs. Thus, in-depth knowledge about how nurses themselves, older people and their relatives describe and experience these issues appears critical. This is particularly the case because being cared for by the right health professionals, as well as receiving fundamental nursing based on needs, values, and preferences (c.f. [39]), can be viewed as an obvious reflection of quality of care. This knowledge can support the development of relevant nursing interventions targeting older people’s fundamentals of care needs while also ensuring the continuity and quality of care delivery within the home- or facility-based care context. Thus, the present scoping review aims to investigate and descriptively summarise previous research about fundamental nursing, its focus (what care needs are described, how is it described and by whom is it described), continuity of care (is it described in relation to fundamental nursing) and possible nursing interventions or activities targeting older people’s fundamentals of care needs in home- or facility-based care.

## Methods

This scoping review was carried out following the steps of Arksey and O’Malley’s [40] methodology and reported in accordance with The Preferred Reporting Items for Systematic Reviews and Meta-Analysis Extension for Scoping Reviews [41]. Scoping reviews are particularly useful when the topic is complex because their methodology enables a systematic charting of findings and supports the identification of research gaps [33, 34]. The latter becomes particularly important when exploring broad topics while also aiming to include all types of research designs, for example, qualitative, quantitative, and mixed methods design. The review protocol was registered a priori with the Open Science framework (https://doi.org/10.17605/OSF.IO/XJ39E). Additionally, a published protocol preceding this review can be located at https://bmjopen.bmj.com/content/13/3/e069798.info.

### Stage 1. Identifying the research question

A modified version of the PICoS framework, for example, population; phenomena of interest; comparison; outcome; and study setting (Table 1), was used to support the development of our research questions and acted as eligibility criteria [42–44].

**Table 1:** PICoS framework

Criteria	Determinants
**P**opulation	Older people (65 years and above), nurses (Table 2) and relatives in a broad understanding
**I**ntervention (phenomenon of interest)	Fundamental nursing care and nursing interventions (Table 2) targeting older people’s fundamental needs
**C**omparison	*Not applicable (NA)*
**O**utcome	Physical, relational, or psychosocial needs Continuity of care Nursing interventions targeting continuity and/or fundamental nursing
**S**tudy setting	Home- and facility-based care (Table 2) for older people

The following research questions were posed to the literature:

i. What type of fundamental nursing (Table 2) is described in the literature as targeting older people’s fundamentals of care needs in home- and facility-based care contexts?
ii. How is fundamental nursing targeting the fundamentals of care described and experienced by key-stake holders (Table 2) in home- and facility-based care contexts?
iii. What fundamental nursing interventions (Table 2) are described in the literature targeting older people’s fundamentals of care needs and/or continuity of care in home- and facility-based care contexts?

**Table 2:** Operationalisation of core concepts in the review

The fundamentals of care are operationalised here as follows:
- Fundamental nursing interventions or activities focusing on physical (i.e., mobility, nutrition, personal care, etc.), psychosocial and relational fundamentals of care needs (i.e., patient involvement, information, emotional well-being, and dignity as well as supporting relatives) [5, 45]
Home- or facility-based care is operationalised here as follows:
- Healthcare delivered over prolonged periods of time in the community, either as home-based care (i.e., home health nursing) or facility-based care (i.e., nursing homes and/or residential aged care facilities (denomination for nursing homes in North America and Oceania) [46–49]
Continuity of care is operationalised after the World Health Organisation’s definition:
- ‘The degree to which a series of discrete healthcare events is experienced by people as coherent and interconnected over time and consistent with their health needs and preferences’ (p. 8) [50]
Here, key stakeholders are operationalised as follows:
- Older people (above 65 years), relatives and nurses
Nurses and/or nursing staff are operationalised as follows:
- Registered and auxiliary nurses (such as, but not limited to, registered nurses, nursing aides, healthcare assistants and personal support workers) [51, 52]
Nursing interventions are operationalised as follows:
- Distinctly articulated and defined nursing interventions and strategies (i.e., models of care, patient care pathways or clinical practice guidelines) with the objective of improving human health [53, 54]

### Stage 2. Identifying relevant studies

To support the identification of relevant studies and be able to decide upon reasonable searches, all core concepts of importance for the topic in focus were carefully operationalised (Table 2). Searches were conducted in PubMed via NIH, CINAHL via EBSCO and PsycInfo via ProQuest. Comprehensive and adapted search strategies (additional file 2) were developed, tested, and evaluated, by the research team together with a librarian. The process of developing relevant search strings begun in PubMed and were conducted in a stepwise iterative manner by the first (OMN) and last author (GB). The first author drafted a tentative search string, conducted an initial screening search. The latter were thereafter discussed and evaluated with the last author before further adjustments were done. Finalised PubMed search strategies then became the main template for the development of search strings in the two remaining databases.

Database-specific headings and medical subject headings were used. Search blocks were applied combined with keywords, synonyms, and the Boolean operators AND/OR [55]. Limits were set to include English written peer-reviewed primary research published between 1 January 2002 to 12 May 2023. The time limit was set based on the fact that long-term care contexts have undergone considerable changes during the past two decades [56].

### Stage 3. Study selection

**Fig. 1:**
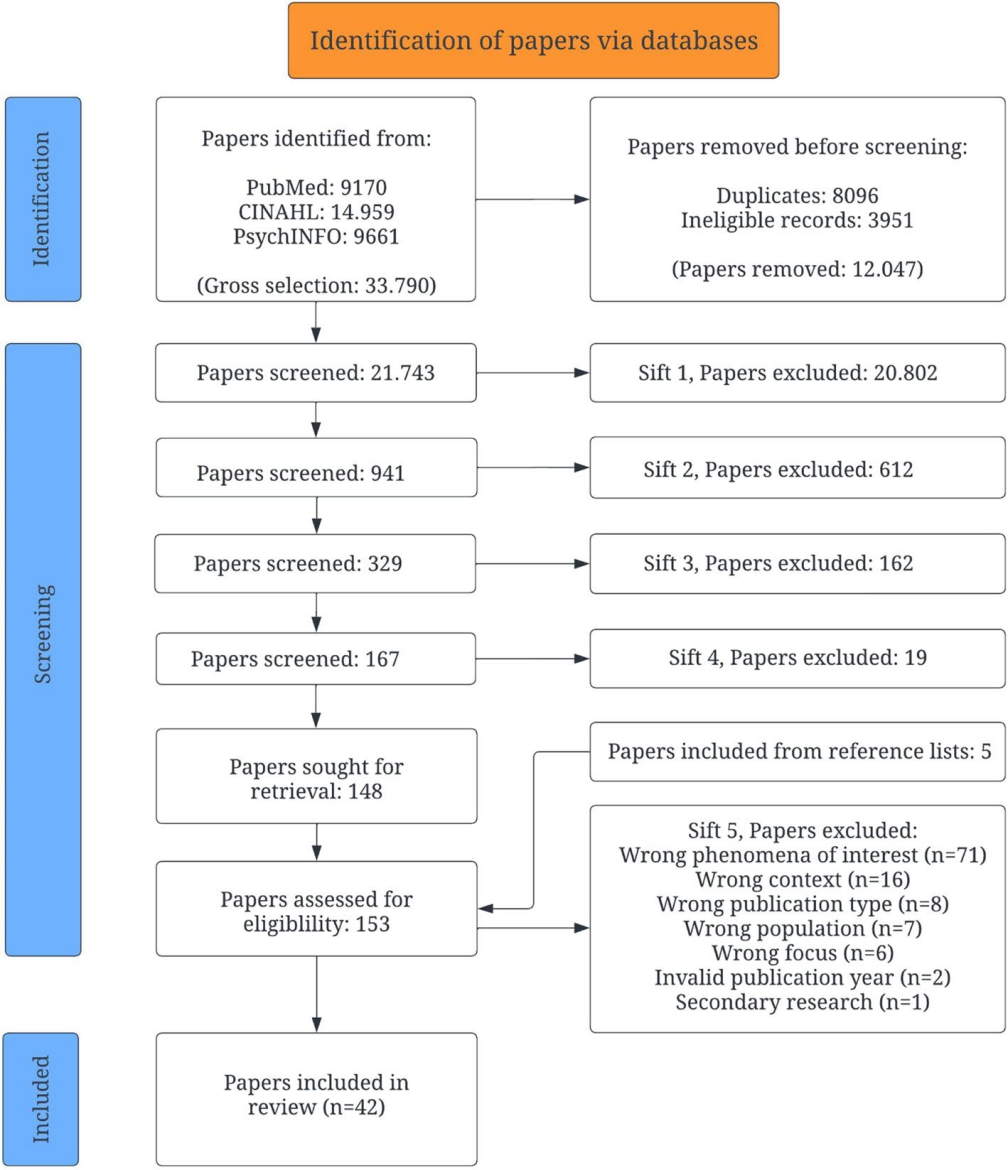
PRISMA flowchart

Eligible publications for each of the three research questions were imported individually and grouped in EndNote by the first author [57]. In EndNote, an initial screening supported the removal of duplicates, editorials, commentaries, and secondary research. The remaining publications were then imported to Rayyan [58]. All authors conducted a joint a title—abstract screening guided by the developed PICoS determinants (Table 1). We screened, independently of each other, 567 papers in pairs to assure an agreement on what to include and exclude. Thereafter, a stepwise title – abstract approach was utilised where the first (OMN) and last author (GB) “sifted” [59] through, in close collaboration, the total numbers of eligible papers for each research question (Fig. 1) [60]. During the whole of this process conflicts were discussed and if necessary, solved by a third reviewer. The process ended with handsearching the reference lists, a backward citation tracking, of the papers evaluated as to be read in full text [61, 62].

### Stage 4. Charting the data

The extraction chart was piloted by the first author (OMN) and cross-checked by the research team (ERG, CO, HA, GB). Hence, a random sample of 10 eligible papers conducted with either qualitative, quantitative, or mixed methods was extracted. The piloting resulted in minor adjustments, i.e., in what order extracted data was presented. Data extraction included country of origin, year, authors, design, aim, method, and results. Additionally, data facilitating an in-depth exploration such as type of home- and facility-based care contexts, whose perspective, type of nurses, descriptions and outcome(s) of nursing interventions were also extracted. The first author (OMN) conducted the initial data extraction in close collaboration with the last author (GB). Thus, full text reading (*n* = 42) and data extraction was done in close collaboration between OMN and GB.

Quality assessments were conducted by the first author (OMN) and the assessments were continuously discussed with the last author (GB). Relevant critical appraisal tools for each individual design, for example, the Critical Appraisal Skills Programme qualitative checklist (CASP) [63] and Mixed Methods Appraisal Tool (MMAT) [64] was used. Assessing included papers quality supported us to identify research of poorer quality. It additionally supported us to formulate ´clear statements about possible knowledge gaps as well as saturated areas not requiring further explorations. The assessment of ethical standards was conducted using Weingarten et al.’s recommendation for evaluating ethics in systematic reviews [65].

### Stage 5. Collating, summarising, and reporting the results

**Fig. 2:**
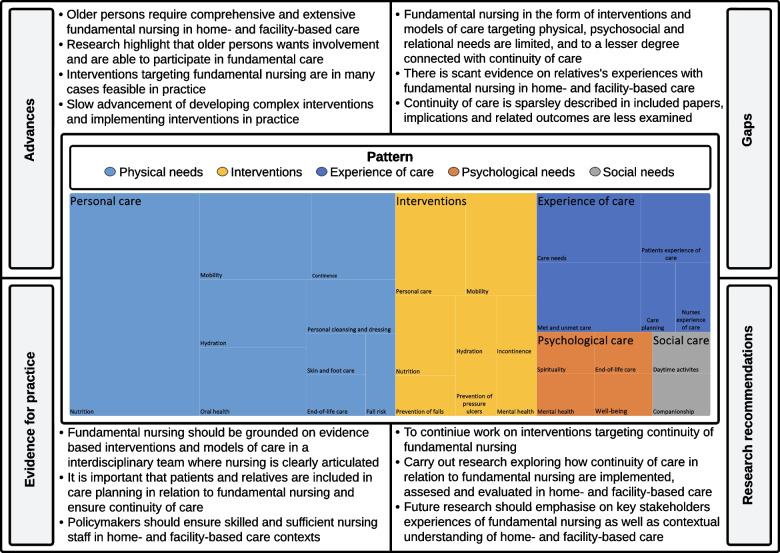
Overview model based on the PAGER framework displaying main results

Extracted data evaluated to answer Q1, Q2 (Additional file 3) and Q3 (Additional file 4) underwent a basic descriptive analysis in accordance with recommendations [66]. The first author (OMN) took the lead in the process of analysis. This entailed repeated readings and summation of content, while the main focus was on the descriptive and manifest content and on organising and categorise extracted data into patterns [67]. The descriptive analysis and the emerging findings was discussed between the first (OMN) and last author (GB) but also in monthly meetings with the rest of the research team (ERG, CO, HA). Extracted data evaluated to answer Q3 was transferred into a table to summarise and describe the key intervention components and outcomes. Key information from each paper were integrated and summarised with the support of the PAGER framework (pattern, advances, gaps, evidence for practice and research recommendations) [67]. This strategy supported the research team to develop an overview model of the main results (Fig. 2) but also to streamline the presentation of complex data i.e. making the main result easier to grasp. Further development of the PAGER framework has been suggested by Bradbury-Jones et al. [67], and we propose that the PAGER framework can support innovative solutions in providing a comprehensive overview of complex results.

## Results

In this scoping review a total of 42 papers were included (Fig. 1). Of these 42 included papers 32 of them was assessed to answer Q1 that is, *“what type of fundamental nursing is described in relation to older people´s needs in home- and facility-based care contexts*” and Q2 that is, *“how fundamental nursing is described and experienced by the key stakeholders in a home- or facility-based care context”* (Additiinal file 3). The characteristics of the latter were that they all in all represented 4,655 participants. Older people (*n* = 3,655 [78.5%]) were in majority with a mean age of 84.4 years old and 63.4% of them were female. The second largest population were registered nurses (*n* = 235), followed by nursing assistants (*n* = 194), relatives (*n* = 161) and non-specified healthcare staff (*n* = 122). Consequently, RNs, nurse assistants and other healthcare staff only made up 11.8% of the participants in the included papers. 24 of the papers represented research conducted in a facility-based care context [68*, 69*, 70*, 71*, 72*, 73*, 74*, 75*, 76*, 77*, 78*, 79*, 80*, 81*, 82*, 83*, 84*, 85*, 86*, 87*, 88*, 89*, 90*], six in a home-based context [91*, 92*, 93*, 94*, 95*, 96*], and two had been conducted in both contexts [97*, 98*]. The included papers represented a variety of research designs; qualitative descriptive design (*n* = 10), cross-sectional (*n* = 6), ethnography (*n* = 4), mixed methods (*n* = 3), observational study (*n* = 2) and qualitative exploratory design (*n* = 2). Five papers represented research designs such as: lifeworld design, participant observations, qualitative emergent case study, prospective cohort, and multi-methods.

Fundamental nursing was described (Q1) as mainly focusing on older people’s different physical care needs. Nutrition—or rather nutritional care activities targeting eating and drinking—was the most frequently described care need [68*, 69*, 74*, 75*, 76*, 82*, 87*, 95*, 97*, 98*, 99*]. Followed by descriptions of personal care such as personal cleansing, dressing, oral care, skin care and foot care [70*, 72*, 83*, 96*], elimination [73*, 77*, 91*] and maintaining mobility and functional ability [78*, 79*, 80*]. Other included papers [84*, 85*, 86*, 88*, 89*, 90*, 92*, 94*] targeted older persons´ fundamental care needs in a more general approach. These focused on medication management, specific nursing procedures such as compression stocking application, wound care, observation (i.e., weight, blood pressure) as well as more advanced and technical nursing such as maintenance of urinary catheter, stoma and feeding tube [94*]. They also described assessment of care needs [88*], end-of-life care [86*] and how older persons prioritise their care needs [90*]. Finally, fundamental nursing also targeted older people’s activities of daily living, social care needs, involvement, and well-being [84*, 85*, 92*]. Fundamental nursing targeting psychosocial and relational needs was to a lesser degree reflected in the literature [71*, 81*, 93*].

Fundamental nursing was described and experienced (Q2) as complex, comprehensive, and taxing. Fundamental nursing affected a broad range of healthcare needs, ranging from physical, psychosocial, and relational, which, in turn, were described as demanding a high skillset and knowledge from the nursing staff situated within complex healthcare organisations. Nurses and older people also described a lack of communication, teamwork, and coordination of care, [68*, 72*, 73*, 79*, 85*, 92*, 97*] which in many cases originated from inadequate support and resources [74*, 75*, 77*, 99*]. The nurses also described older people as frailer and more dependent than before, which resulted in an increased need for skills, knowledge, and support [89*, 98]. Older people frequently described a gap between the provided fundamental nursing and their perceived need for support [71*, 81*, 84*, 86*, 88*, 92*]. Relatives reported more unmet needs than the nursing staff did [85*]. A recurrent pattern related to the challenges of implementing evidence-based and effective nursing interventions targeting and meeting older peoples´ fundamental needs was also described by the nurses [68*, 69*, 71*, 72*, 77*, 79*, 82*, 83*, 89*, 95*, 97*, 98*]. Descriptions also highlighted a lack of both sufficient and adequately trained nursing personnel but also its relationship with less-than-optimal fundamental nursing within these contexts [73*, 79*, 80*, 89*, 90*, 93*, 94*, 97*, 98*, 99*]. Moreover, several papers described understaffed wards [73*, 79*, 80*, 89*, 90*, 93*, 97*, 98*, 99*]. Consequently, RN frequently described that they task-shifted and delegated fundamental nursing activities to healthcare assistants and personal support workers. Thus, resulting in that they were described to contribute less to fundamental nursing [68*, 72*, 73*, 91*, 96*]. The nurses additionally described they felt underequipped in relation to attending psychosocial care needs [71*, 81*, 85*, 86*, 93*] because fundamental nursing was first and foremost described as being oriented towards physical care needs, and as a result, psychosocial and relational needs were at risk of being less than optimal [71*, 85*, 86*, 89*, 93*].

Older people described being dependent on fundamental nursing as challenging. Needing to rely on other people to maintain otherwise daily activities, such as nutritional needs, being mobile and taking care of personal needs, was described as being in a vulnerable situation [69*, 70*, 75*, 78*]. In certain scenarios, older people described how being dependent on others for their fundamental nursing needs could be amplified through degrading situations, from being left on the toilet for extended periods [70*], not having access to the kitchen limiting access to refreshments [69*], not being involved in nutritional care [68*, 75*, 82*, 95*] and experiencing that calls for help were delayed and unattended [90*] or finding that nursing staff were rushing mobility care [78*]. Older persons described that they were not adequately cared for, involved, or invited to participate using their remaining strength and function to be engaged in their fundamentals of care [69*, 71*, 72*, 74*, 75*, 78*]. It is worth nothing that older peoples´ perspective of fundamental nursing in relation to Q1 and Q2 were represented in about 56% of the included papers [69*, 70*, 76*, 77*, 78*, 79*, 82*, 83*, 84*, 85*, 86*, 87*, 88*, 90*, 92*, 94*, 95*, 99*] whereas the RNs perspective were represented in about 25% of them [71*, 72*, 81*, 89*, 91*, 93*, 97*, 98*]. In four of the included papers, a minor percentage of the population were under 65 years [69*, 78*, 79*, 82*]. These papers were included based on the relevance of the overall population, phenomena of interest and ability to answer the research questions.

Of the 42 included papers in this scoping review 10 of them was assessed to answer Q3 that is*,” fundamental nursing interventions targeting older people’s fundamental needs or their continuity of care in home- or facility-based care contexts”* (Additional file 4). The characteristics of the latter were that they all in all represented 1,741 participants and older people (*n* = 1,119, [64.2%]) were also in majority here. Their mean age was 84.8 years old and 80.2% of them were female. The second largest population here were nursing assistants (*n* = 291), followed by non-specified care staff (*n* = 93), and RNs (*n* = 33). RNs, nurse assistants and other healthcare staff made up about 37.2% of the participants in the included papers assessed to answer Q3. Hence, nurses’ perspective on Q3 were represented in 40% of the included papers [100*, 101*, 102*, 103*] and older peoples’ perspective were represented in about 60% of the papers [104*, 105*, 106*, 107*, 108*, 109*]. Eight of the papers represented research conducted in a facility-based care context [100*, 101*, 102*, 104*, 105*, 106*, 107*, 108*], one within home-based care [109*] and one had been conducted within both contexts [103*]. Different experimental designs (*n* = 8) were used to evaluate nursing interventions [101*, 102*, 104*, 105*, 106*, 107*, 108*, 109*], while two studies used qualitative methods to assess intervention development [100*, 103*].

Nursing interventions targeting fundamental needs or continuity of care (Q3) were first and foremost described as focusing on physical care needs, such as preventing falls and pressure ulcers and increased nutritional intake [103*], nutrition and hydration [101*, 102*, 105*], mobility [104*], individual tailored activity [109*], mobility, continence care and hydration [106*], mobility and continence care [107*], foot care [108*] and person-centred models of care targeting fundamental needs [100*]. One paper [100*] described a more comprehensive approach, including physical, psychosocial and relational needs using a human-rights perspective [100*]. The findings indicated that interventions focusing on fundamental nursing were largely feasible in practice and had favourable [102*, 106*, 109*] or moderate results [101*, 103*, 104*, 105*, 107*, 108*]. However, the long-term effects of interventions were difficult to detect because a majority of the 10 included papers assessed to answer Q3 described either that any positive gain from the intervention dropped back to baseline after the evaluation period [101*, 104*] or the effects of the intervention were impaired because of barriers on policy and system level [102*, 103*, 106*, 107*].

None of the included papers, published after the first release of the Medical Research Councils Frameworks for Interventions for Complex interventions [110], used frameworks or guidelines for intervention development [111] or frameworks for developing and evaluating complex interventions [112]. We were not able to identify any fundamental nursing interventions focusing on relational and psychosocial care needs alone where a reasonable primary or secondary outcome could have been continuity of care. Neither did we identify any fundamental nursing interventions targeting older people’s continuity of care.

## Critical appraisal

Following the guidelines of CASP [63], MMAT [64] and Weingarten et al. [65], all papers were screened for methodological and ethical standards (Table 3). For papers assessed with CASP [68*, 69*, 70*, 71*, 72*, 73*, 74*, 75*, 76*, 77*, 78*, 79*, 80*, 81*, 91*, 92*, 93*, 97*, 99*, 100*, 103*], the results indicated that the overall quality is acceptable. However, only 23.8% [68*, 69*, 78*, 80*, 100*] clearly declared the relationship between researcher and participant, and only 28.5% clearly declared ethical considerations [76*, 78*, 80*, 91*, 97*, 100*]. For papers assessed with MMAT [82*, 83*, 84*, 85*, 86*, 87*, 88*, 89*, 90*, 94*, 95*, 96*, 98*, 101*, 102*, 104*, 105*, 106*, 107*, 108*, 109*], the quality was overall acceptable; however, for quantitative descriptive studies [82*, 83*, 84*, 85*, 86*, 87*, 88*, 94*, 95*, 96*], half of the papers did not clearly state if the risk of nonresponse bias was low [83*, 84*, 85*, 86*, 95*]. Finally, in relation to ethical assessment of all papers (*N* = 42), the results ranged from poor (0 of 5) to excellent reporting (5 of 5) (M = 3.04, Mdn = 3, mode = 4). A less reported topic was the declaration of adequate data protection because only 7.1% clearly provided a description of how research data were handled, stored, and protected [68*, 91*, 94*].

**Table 3:** Critical appraisal

No #	CASP	MMAT	Ethics	No #	CASP	MMAT	Ethics	No #	CASP	MMAT	Ethics
Blomberg et al., 2021 [97*]	8/9	NA	3/5	Borglin et al., 2020 [91*]	8/9	NA	4/5	Christensson et al., 2003 [101*]	NA	7/7	3/5
Ernsth Bravell et al., 2021 [92*]	7/9	NA	4/5	Frändin et al., 2016 [104*]	NA	6/7	4/5	Greene et al., 2021 [69*]	8/9	NA	4/5
Grøndahl & Aagard, 2016 [82*]	NA	6/7	4/5	Hansen et al., 2017 [93*]	7/9	NA	4/5	Holmberg et al., 2019 [70*]	7/9	NA	3/5
Housley et al., 2008 [83*]	NA	6/7	0/5	Hunter & Levett-Jones, 2010 [89*]	NA	6/7	2/5	Ibrahim et al., 2020 [100*]	8/9	NA	4/5
King et al., 2020 [96*]	NA	6/7	4/5	Kuo et al., 2019 [71*]	7/9	NA	4/5	Lannering et al., 2017 [103*]	7/9	NA	2/5
Lindqvist et al., 2013 [72*]	7/9	NA	3/5	Ludlow et al., 2021 [90*]	NA	4/7	2/5	Mather & Bakas, 2002 [73*]	7/9	NA	2/5
Mentes et al., 2006 [74*]	7/9	NA	1/5	Murphy et al., 2017 [75*]	7/9	NA	3/5	Martin et al., 2002 [84*]	NA	6/7	1/5
Mentes & Culp, 2003 [105*]	NA	5/7	1/5	Næss et al., 2017 [94*]	NA	6/7	5/5	Orrell et al., 2008 [85*]	NA	6/7	5/5
Pasman et al., 2003 [76*]	8/9	NA	3/5	Parsons et al., 2013 [109*]	NA	6/7	4/5	Persenius et al., 2008 [98*]	NA	4/7	2/5
Van der Ploeg et al., 2013 [88*]	NA	6/7	4/5	Reynolds et al., 2002 [86*]	NA	6/7	3/5	Schnelle et al., 2002 [106*]	NA	7/7	3/5
Simmons & Ouslander, 2005 [107*]	NA	3/7	3/5	Simmons & Patel, 2006 [99*]	7/9	NA	4/5	Sjögren Forss et al., 2018 [68*]	8/9	NA	5/5
Soini et al., 2006 [95*]	NA	6/7	2/5	Stolt et al., 2001 [108*]	NA	7/7	4/5	Suominen et al., 2005 [87*]	NA	6/7	2/5
Taunton et al., 2005 [77*]	7/9	NA	1/5	Taylor et al., 2014a [78*]	9/9	NA	3/5	Taylor et al., 2014b [79*]	6/9	NA	4/5
Törmä et al., 2018 [102*]	NA	6/7	3/5	Wu et al., 2013 [80*]	9/9	NA	3/5	Ødbehr et al., 2015 [81*]	7/9	NA	4/5

## Discussion

This scoping review aimed to investigate and descriptively summarise previous research about fundamental nursing, its focus (what care needs are described, how is it described and by whom is it described), continuity of care (is it described in relation to fundamental nursing) and possible nursing interventions or activities targeting older people’s fundamentals of care needs in home- or facility-based care. The main results (Fig. 2) suggested that fundamental nursing primarily focused on physical needs and less attention was described towards relational- and psychosocial needs. Nursing interventions targeting all aspects of fundamental nursing and/or continuity of care was to a little degree reflected in the included data material. The results are further discussed based on the PAGER framework in Fig. 2 [113].

### Pattern

The pattern of the included papers suggested that the scientific literature describing fundamental nursing (Q1), the experience and descriptions of fundamental nursing (Q2), interventions or activities targeting fundamental nursing and continuity of care (Q3) were in most cases focusing on individual physical needs. In many papers, fundamental nursing was described to point towards fragmented and suboptimal fundamental nursing in home- and facility-based care, in line with previous literature reviews [10, 114–116] and primary research studies [117, 118]. The pattern of fragmented care could also be put in relation to one of the recurring descriptions from the analysis, that is, the lack of both sufficient and adequately trained nursing personnel and relationship to less-than-optimal fundamental nursing within home- and facility-based care [68*, 69*, 71*, 72*, 73*, 75*, 79*, 80*, 83*, 89*, 90*, 93*, 94*, 97*, 98*, 107*]. Previous research has pointed towards strong evidence supporting the correlation between nurse staffing, competence and patient mortality in specialised healthcare contexts [119, 120] and the relationship between missed nursing care, adverse events and patient safety [31]. Within facility-based care, White et al. [121] found that RNs portrayed high levels of nurse burnout and job dissatisfaction. Research has pointed towards a relation between lack of access to resources and missed nursing care, which resulted in negative physical outcomes, that is, pressure ulcers, unnecessary use of antipsychotic medication and unplanned hospital admissions, as well as psychosocial and relational outcomes, that is, comforting and talking with older people and involving them as well as their relatives [122]. Results indicated that fundamental nursing, mostly related to physical care, largely overlooked psychosocial and relational aspects, and lacked comprehensive models of care focusing fundamental nursing and continuity of care.

### Advances

The literature in this scoping review can be placed within the general discourse of nursing. Using Henderson’s nursing theory [1], both as a historical reference and theoretical perspective, the results suggest that the included papers remain primarily focused on physical needs because we could find few advancements in other aspects of fundamental nursing, such as the involvement of older people and their relatives and engagement in activity, which Henderson also saw as principles of fundamental nursing [1]. Hence, the results indicate slow advancement in theoretical, empirical, and interventional development focusing on fundamental nursing and continuity of care. As a result, nurses have few evidence-based models of care targeting fundamental nursing and continuity of care to implement within home- and facility-based care contexts. The modest state of research on fundamental nursing has been discussed elsewhere [10, 116].

Later theoretical developments on nursing theory [123] have adopted a more comprehensive approach that could be beneficial because the results demonstrated that older persons have complex and comprehensive fundamental nursing care needs. Only a few papers have taken a more comprehensive approach to nursing interventions [100*, 103*, 104*, 107*]. However, the identified interventions can be viewed as narrow when compared with fundamental nursing, which ideally should target relational, psychosocial and physical needs [5]. The lack of advancement generates several key uncertainties and knowledge gaps concerning nursing interventions targeting fundamental needs and continuity of care in the literature.

### Gaps

A number of key uncertainties and knowledge gaps were identified. Most central is that the relationship between fundamental nursing and continuity of care is poorly described in the literature. Hence, any outcomes of continuous fundamental nursing targeting physical, relational and psychosocial needs are less understood. Second, as the results suggest that descriptions of fundamental nursing as both fragmented and complex is not uncommon it is reasonable to conclude that both the continuity- and quality of care might be infringed among older people in the home- and facility-based care context. Lacking teamwork, an optimal communication, and coordination of care [68*, 72*, 73*, 79*, 85*, 92*, 97*] together with the already earlier mentioned description of understaffed, underequipped, and under-resourced home- and facility-based care contexts further supports such conclusions. However, less is known because the research focusing on fundamental nursing pinpoints a lack of conducted research targeting home- and facility-based care contexts [124]. The scarcity of nursing research focusing on continuous and comprehensive fundamental nursing gives the incentive to explore both home- and facility-based contexts and older persons’ fundamental nursing needs and their relatives’ experiences of fundamental nursing or lack thereof. The experiences of the latter were to a little degree reflected in the studies included in this scoping review because relatives represented only 4.25% of the total population, despite experiencing a tremendous burden and responsibility of informal care among older people [125].

### Evidence for practice

Fundamental nursing should be grounded on evidence-based interventions and models of care, based on the involvement of the older person and their relatives in establishing a coherent and interconnected fundamental nursing and consistent with the older person’s needs and preferences over time [50]. Such care is more likely to promote safe, timely, effective, efficient, equitable and person-centred [126] fundamental nursing with the quality older persons should expect from home- and facility-based care [22] in a dignifying manner [37, 38]. One possible way to increase the quality of fundamental nursing in home- and facility-based care is to employ and train highly skilled nursing staff. However, as the Committee on the Quality of Care in Nursing Homes stated, ‘low staff salaries and benefits combined with inadequate training has made the nursing home a highly undesirable place of employment’ [22] (p. 3); as a result, the much needed nursing workforce is looking elsewhere for employment [127–129]. Hence, alleviation of complex challenges is not only within the remit and competence of nursing staff; the results also point towards shortcomings on policy and system levels.

### Research recommendations

Little research has been done on models of care, guidelines and frameworks that could support nurses in promoting fundamental nursing (Q3) in relation to the constituent parts of continuity of care, that is, fundamental nursing, which is experienced as coherent, interconnected over time and consistent with older peoples’ needs and preferences [50]. Although earlier research has targeted nurse-led interventions [130] and continuity of care [26, 131–133], we could not detect interventions focusing on continuous fundamental nursing addressing older people in home- and facility-based care. Thus, our results have implications in relation to research. First, because nurses have few options of interventions focusing on fundamental nursing and continuity of care, the results underscore the need for intervention development aiming to support nurses in promoting a comprehensive approach to fundamental nursing, ensuring that the individual’s needs are regularly assessed and evaluated to ensure an optimal and continuous mode of fundamental nursing. Second, because there is a scarcity of research targeting fundamental nursing beyond older peoples’ single obvious physical needs e.g. nutrition, mobility and hygiene. Whereas other vital parts of fundamental nursing such as relational- and psychosocial needs are less well researched. On the other our findings implies that RNs perspectives of fundamental nursing is scant. This is noteworthy as non-registered assistants, although of vital importance in care, should be conceived as; “the operational arm of registered nurses (RNs) carrying out nursing behaviour under supervision and leadership from RNs” [2] (p. 149). Thus, there is a need to further explore current nursing practices to examine in more detail fundamental nursing and continuity of home- and facility-based care.

## Methodological strengths and limitations

This scoping review has some strengths, such as the development of a wide search strategy that accommodates the PICoS of this review. The search strategy was developed with sensitivity to detect interchangeable use of vocabulary concerning home- and facility-based care, nursing staff, continuity of care, interventions, and fundamental nursing. To ensure that we had developed an optimised search strategy, we piloted and revised the search strategies, which were accompanied by meetings within the research team and consulting research librarians. However, valuable papers might not have been detected by our search strategy. A limitation and departure from the original methodology [40] on scoping reviews is the discarding of grey literature. The inclusion of grey literature (and other languages) could potentially expand our results, identifying practical guidelines and pathways in relation to fundamental nursing among older people in home- and facility-based care contexts and providing a deeper contextual understanding. However, this was not within the aim of the present study. Our choice to not exclude included papers, based on their quality assessment can be viewed as both a caution for the interpretation of our findings but also as a strength as even papers with limited quality can provide a valid rationale as a guidance as to where more research is required or to specific methodological recommendations for future research (c.f. [61]).

A central component of the present scoping review is its alignment with the Medical Research Council’s (MRC) framework for developing complex interventions [112]. This scoping review acts as the initial stage based on the MRC framework, which emphasises the development or identification of interventions, characteristics, and target population, as well as taking into consideration core elements (i.e., considering context, identifying uncertainties and stakeholder viewpoints) [112]. As such, guiding future research by informing appropriate research questions and perspectives [112]. A scoping review supports intervention development by identifying what is already known and pinpointing evidence gaps [67]. This feature can strengthen quality [112], inform planning of future research, prevent research waste [134], and ensure value through justifiable research priorities [135].

## Conclusion

The present study has provided a summation extracted from a large body of scientific literature based on 42 included papers. The results suggested a fragmented and compartmentalised body of scientific literature as fundamental nursing was mainly described in relation to physical care needs, dominantly consisting of nutrition, mobility, hydration, oral health, and personal care needs essentially conducted within facility-based care contexts. Interventions and activities focusing on fundamental nursing primarily focused on one fundamental need at a time, mainly within the physical domain. Embedded strategies within nursing interventions were, to a little degree, targeting relational- or psychosocial needs where continuity of care could act as a possible outcome. This was reflected by older people as they described less attention to relational and psychosocial needs as opposed to physical care needs. Stakeholders’ viewpoints suggested that contextual factors, staffing, resources, and competence were the driving factors influencing the quality of fundamental nursing. Further research is needed to develop interventions, departing from the MRC framework [112] focusing on comprehensive and continuous fundamental nursing because the older population is increasing and the demand for fundamental nursing in home- and facility-based care contexts will continue to rise in the coming years.

### Supplementary Information

**Additional file 1.** PRISMA-ScR checklist. 

**Additional file 2.** Search strategy PubMed.

**Additional file 3.** Overview of papers answering questions one and two.

**Additional file 4.** Overview of papers answering question three.

## Data Availability

The datasets supporting the conclusions of this article are available at request.

## Abbreviations

CASP: Critical appraisal tool
MeSH: Medical subject heading
MMAT: Mixed methods appraisal tool
NIH: National Institutes of Health
PAGER: Patterns, advances, gaps, evidence for practice and research recommendations
PICoS: Population, phenomena of interest, comparison, outcome and study setting
PRISMA: Preferred reporting items for systematic review and meta-analysis
RN: Registered nurse
WHO: World Health Organisation
