# Supplementary material for: What about the fundamentals of nursing—its interventions and its continuity among older people in need of home- or facility-based care: a scoping review
Source: BMC Nurs. 2024 Jan 22;23:59. doi: 10.1186/s12912-023-01675-1 (PMC10801980; doi:10.1186/s12912-023-01675-1)
Supplement: Supplementary file 2 — Additional file 2. Search strategy PubMed. [file 12912_2023_1675_MOESM2_ESM.docx]

Additional file 2 - Search strategy PubMed, last updated 12.5.23

| Nr | Population - Older people | Results |
| --- | --- | --- |
| #1 | Aged [MeSH Terms] | 3.446.470 |
| #2 | Aged [Title/abstract] | 719.593 |
| #3 | Elder* [Title/abstract] | 307.140 |
| #4 | Old [Title/abstract] | 1.246.062 |
| #5 | Seniors [Title/abstract] | 8.924 |
| #6 | Senior [Title/abstract] | 41.810 |
|  |  |  |
| #7 | ((((((#1) OR (#2)) OR (#3)) OR (#4)) OR (#5)) OR (#6) | 5.000.409 |
|  |  |  |

| Nr | Population - Relatives | Results |
| --- | --- | --- |
| #8 | Family [MeSH term] | 365.909 |
| #9 | Spouse [MeSH term] | 11.545 |
| #10 | Husband* [Title/abstract] | 21.339 |
| #11 | Wife [Title/abstract] | 6.915 |
| #12 | Wives [Title/abstract] | 5.711 |
| #13 | Partner* [Title/abstract] | 214.691 |
| #14 | Daught* [Title/abstract] | 28.683 |
| #15 | Son [Title/abstract] | 12.130 |
| #16 | Relatives [Title/abstract] | 66.539 |
| #17 | Significant other* [Title/abstract] | 4.742 |
| #18 | Next of kin [Title/abstract] | 1.752 |
|  |  |  |
| #19 | (((((((((((#8) OR (#9)) OR (#10)) OR (#11)) OR (#12)) OR (#13) OR (#14)) OR (#15) OR (#16)) OR (#17) OR (#18)) | 666.109 |
|  |  |  |

| Nr | Population - Nursing staff | Results |
| --- | --- | --- |
| #20 | Nursing staff [MeSH Terms] | 69.753 |
| #21 | Nurse*[MeSH term] | 161.855 |
| #22 | Licenced practical nurse* [MeSH terms] | 941 |
| #23 | Nursing staff [Title/abstract] | 14.590 |
| #24 | Nurse* [Title/abstract] | 320.499 |
| #25 | Licenced practical nurse [Title/abstract] | 753 |
| #26 | Registered nurse* [Title/abstract] | 14.304 |
| #27 | Licensed practice nurse* [Title/abstract] | 42 |
| #28 | Registered practical nurse* [Title/abstract] | 77 |
| #29 | Licensed vocational nurse* [Title/abstract] | 30 |
| #30 | Nurse specialist* [Title/abstract] | 4.327 |
| #31 | Health care professional* [Title/abstract] | 3.936 |
| #32 | Practicing nurse* [Title/abstract] | 695 |
| #33 | Professional nurs* [Title/abstract] | 2.066 |
| #34 | Associate nurs* [Title/abstract] | 57.361 |
| #35 | Formal caregiver [Title/abstract] | 340 |
| #36 | Nurs* assistant* [Title/abstract] | 10.345 |
| #37 | Nursing aide* [Title/abstract] | 299 |
| #38 | Health care assistant [Title/abstract] | 154 |
| #39 | Care provider* [Title/abstract] | 69.385 |
|  |  |  |
| #40 | (((((((((((((#20) OR (#21)) OR (#22)) OR (#23)) OR (#24)) OR (#25) OR (#26)) OR (#27) OR (#28) OR (#29)) OR (#30)) OR (#31) OR (#32)) OR (#33) OR (#34) OR (#35) OR (#36) OR (#37) OR (#38) OR (#39) | 500.218 |
|  |  |  |

| Nr | Phenomenon of Interest - Continuity of care | Results |
| --- | --- | --- |
| #41 | Continuity of patient care [MeSH term] | 287.743 |
| #42 | Continuity of nursing care [Title/abstract] | 9 |
| #43 | Continuity of care [Title/abstract] | 8.905 |
| #44 | Care continuity [Title/abstract] | 732 |
| #45 | Informational continuity [Title/abstract] | 89 |
| #46 | Relational continuity [Title/abstract] | 179 |
| #47 | Interpersonal continuity [Title/abstract] | 48 |
| #48 | Longitudinal continuity [Title/abstract] | 89 |
| #49 | Management continuity [Title/abstract] | 79 |
|  |  |  |
| #50 | (((((((((((((#41) OR (#42)) OR (#43) OR (#44)) OR (#45) OR (#46)) OR (#47)) OR (#48) OR (#49)) | 293.476 |
|  |  |  |

| Nr | Phenomenon of Interest - Care & nursing | Results |
| --- | --- | --- |
| #51 | Nursing care [MeSH term] | 141.272 |
| #52 | Nursing [MeSH term] | 263.153 |
| #53 | Patient* care need* [Title/Abstract] | 352 |
| #54 | Basic care [Title/Abstract] | 752 |
| #55 | Essence of care [Title/Abstract] | 332 |
| #56 | Essential care [Title/Abstract] | 323 |
| #57 | Fundamental* care [Title/Abstract] | 159 |
| #58 | Eldercare [Title/Abstract] | 517 |
| #59 | Elderly care [Title/Abstract] | 1.853 |
| #60 | Geriatric care [Title/Abstract] | 2.131 |
| #61 | Care aged person* [Title/Abstract] | 320 |
| #62 | Care older person* [Title/Abstract] | 5.915 |
| #63 | Old age assistance [Title/Abstract] | 31 |
| #64 | Care depen* [Title/Abstract] | 809 |
| #65 | Care need* [Title/Abstract] | 18.657 |
| #66 | Physical care need* [Title/Abstract] | 41 |
| #67 | Basic nursing need* [Title/Abstract] | 19 |
| #68 | Physiological need* [Title/Abstract] | 881 |
| #69 | Biological need* [Title/Abstract] | 225 |
| #70 | Psychosocial need* [Title/Abstract] | 2.227 |
| #71 | Physical care [Title/Abstract] | 758 |
| #72 | Advanced care [Title/Abstract] | 1.200 |
| #73 | Care support [Title/Abstract] | 2.356 |
| #74 | Compassionate care [Title/Abstract] | 1.244 |
| #75 | Core nursing [Title/Abstract] | 146 |
|  |  |  |
| #76 | ((((((((((((((#51) OR (#52)) OR (#53)) OR (#54)) OR (#55)) OR (#56) OR (#57)) OR (#58) OR (#59)) OR (#60) OR (#61) OR (#62)) OR (#63) OR (#64)) OR (#65) OR (#66) OR (#67) OR (#68)) OR (#69) OR (#70) OR (#71) OR (#72) OR (#73) OR (#74) OR (#75) | 335.666 |
|  |  |  |

| Nr | Phenomenon of Interest - Models, frameworks, and interventions of care | Results |
| --- | --- | --- |
| #77 | Nursing model [MeSH term] | 12.238 |
| #78 | Nursing process [MeSH term] | 86.604 |
| #79 | Critical pathway [MeSH term] | 7.759 |
| #80 | Practice guideline* [Title/abstract] | 33.917 |
| #81 | Care model* [Title/abstract] | 10.676 |
| #82 | Nursing framework* [Title/abstract] | 125 |
| #83 | Care framework* [Title/abstract] | 699 |
| #84 | Nursing intervention* [Title/abstract] | 7.925 |
| #85 | Nurse-led [Title/abstract] | 4.973 |
| #86 | Patient care pathway* [Title/abstract] | 276 |
| #87 | Patient pathway* [Title/abstract] | 655 |
| #88 | Care pathway [Title/abstract] | 4.098 |
| #89 | Clinical pathway* [Title/abstract] | 4.655 |
| #90 | Clinical recommendation* [Title/abstract] | 4.168 |
| #91 | Care path [Title/abstract] | 265 |
| #92 | Care guideline [Title/abstract] | 375 |
| #93 | Care recommendation* [Title/abstract] | 1.260 |
| #94 | Clinical practice guideline* [Title/abstract] | 20.079 |
|  |  |  |
| #95 | (((((((((((((((((((((((((((((((((#77) OR (#78)) OR (#79) OR (#80) OR (#81)) OR (#82) OR (#83)) OR (#84) OR (#85) OR (#86) OR (#87) OR (#88)) OR (#89) OR (#90) OR (#91) OR (#92) OR (#93) OR (#94) | 166.647 |
|  |  |  |

| Nr | Context - Long term care, home healthcare, home nursing, and other care facilities | Results |
| --- | --- | --- |
| #96 | Long term care [MeSH term] | 28.359 |
| #97 | Housing [MeSH term] | 36.611 |
| #98 | Nursing home* [MeSH term] | 44.190 |
| #99 | Housing for the elderly [MeSH term] | 1.652 |
| #100 | Home health nursing [Title/abstract] | 199 |
| #101 | Home health services [Title/abstract] | 580 |
| #102 | Community health services [Title/abstract] | 1.651 |
| #103 | Primary health care [Title/abstract] | 33.557 |
| #104 | Primary care [Title/abstract] | 143.435 |
| #105 | Community care [Title/abstract] | 5.770 |
| #106 | Community care services [Title/abstract] | 198 |
| #107 | Municipal care [Title/abstract] | 106 |
| #108 | Home care [Title/abstract] | 21.918 |
| #109 | Home dwelling [Title/abstract] | 775 |
| #110 | Home healthcare [Title/abstract] | 1.632 |
| #114 | Home and community-based care [Title/abstract] | 544 |
| #115 | Home and community-based services [Title/abstract] | 764 |
| #116 | Longitudinal care [Title/abstract] | 350 |
| #117 | Assisted living [Title/abstract] | 2.846 |
| #118 | Independent living [Title/abstract] | 3.471 |
| #119 | Sheltered accommodation [Title/abstract] | 87 |
| #120 | Sheltered housing [Title/abstract] | 193 |
| #121 | Residential care [Title/abstract] | 4.343 |
| #122 | Care home [Title/abstract] | 3.143 |
| #123 | Special accommodation [Title/abstract] | 27 |
| #124 | Assisted living facilities [Title/abstract] | 708 |
| #125 | Residential aged care facilit* [Title/abstract] | 964 |
|  |  |  |
| #126 | (((((((((((((((((((((((((((((((#96)) OR (#97) OR (#98)) OR (#99)) OR (#100)) OR (#101) OR (#102)) OR (#103)) OR (#104) OR (105)) OR (#106) OR (#107)) OR (#108) OR (#109) OR (#110)) OR (#111) OR (#112)) OR (#113) OR (#114) OR (#115) OR (#116) OR (#117)) OR (#118) OR (#119) OR (#120) OR (#121) OR (#121) OR (#122) OR (#123) OR (#124) OR (#125) | 306.748 |
|  |  |  |

| Research question | Search string combination | Results |
| --- | --- | --- |
| 1. What type of fundamental nursing (Box 1) is described in the literature as targeting older people’s fundamentals of care needs in home- and facility-based care contexts? 2. How is fundamental nursing targeting the fundamentals of care described and experienced by key-stake holders (Box 1) in home- and facility-based care contexts? | 7 OR 19 OR 40 AND 76 AND 126 + 2002-2022 + English | 7.943 |
| 1. What fundamental nursing interventions (Box 1) are described in the literature targeting older people’s fundamentals of care needs and/or continuity of care in home- and facility-based care contexts? | 7 AND 76 AND 95 AND 126 + 2002-2022 + English | 1.155 |
|  | 7 AND 50 AND 95 AND 126 + 2002-2022 + English | 459 |
|  | Total | 9.557 |
