# Supplementary material for: What about the fundamentals of nursing—its interventions and its continuity among older people in need of home- or facility-based care: a scoping review
Source: BMC Nurs. 2024 Jan 22;23:59. doi: 10.1186/s12912-023-01675-1 (PMC10801980; doi:10.1186/s12912-023-01675-1)
Supplement: Supplementary file 3 — Additional file 3. Overview of papers answering questions one and two. [file 12912_2023_1675_MOESM3_ESM.docx]

| **No** | **Author, year, and country** | **Aim** | **Focus of Fundamental Nursing** | **Setting and Participants** | **Design** | **Vital findings** |
| --- | --- | --- | --- | --- | --- | --- |
| 98 | Blomberg et al., 2020 Sweden | To describe healthcare professionals´ understanding and views of mealtime experience of older persons in municipal care | Nutrition | - N=52, nurse assistants (n=30), RNs (n = 15), occupational therapists (n=7). - Both home- and facility-based care | Qualitative descriptive | Social and psychological needs are integral for optimal nutritional care. Results indicated that nutrition involves a complex set of needs relating to each individual’s unique needs. Lack of teamwork sustained fragmented care |
| 92 | Borglin et al., 2019 Sweden | Illuminate nurses experience of continence care for older people receiving home care | Continence | - N=11, RN (Mean age 49 years, working experience, median 7) - Home-based care | Qualitative descriptive | If RNs delegated continence care, nursing assistants should be supervised and guided. Results indicated that a stepped-care process based on integrated care and systematic nursing assessments facilitate optimal continence care. RNs practiced containment rather than treatment |
| 93 | Bravell et al., 2021 Sweden | Examine older adults’ experiences and perceptions of receiving care and services in their homes | Patient experience of care | - N=29, older persons (Mean age 79 years, age range 66-91 years, median 80) - Home-based care | Qualitative descriptive | Older persons experienced that they were not adequately involved in care, and many older persons described that they were not informed. Results indicate poor informational and relational continuity |
| 70 | Greene et al., 2021 UK | Describe the experience of hydration care for nursing home residents living with dementia | Hydration | - N=22, older persons (Age range 59-101 years) - Facility-based care | Ethnography | Organisation of hydration care was built in around specific activities, as such, these systems also sustained inflexible routines with the outcome of older persons to a lesser degree having access to fluid outside mealtimes |
| 83 | Grøndahl & Aagaard, 2016 Norway | Explore how residents in nursing homes perceive their participation in activities in relation to food and meals, and to identify possible factors that influence their involvement | Nutrition | - N=204, older people (83.3% were 76 years or older, 74% female) - Facility-based care | Cross-sectional | Older persons described how they seldom were included in meal-planning. 90% of the sample did not participate in food preparation and clean-up. 4% felt they could choose when to eat. Results indicated that easier access to food could increase the resident’s control and choice of food |
| 94 | Hansen et al., 2017 Norway | Explore how the psychosocial needs of home-dwelling, older persons with dementia were received, emphasized, and met by home care services, based on descriptions by health care providers | Psychosocial | - N=24, 10 RNs, 14 assistant nurses (100% female) - Home-based care | Qualitative descriptive | Physical and psychosocial needs were seen as separate. Physical care was the main focus, consequently, psychosocial care was seen as the relative’s responsibility or provided by day-care centres. Results indicated need for increased knowledge in relation to psychosocial needs |

| **No** | **Author, year, and country** | **Aim** | **Focus of Fundamental Nursing** | **Target group and setting** | **Design** | **Vital findings** |
| --- | --- | --- | --- | --- | --- | --- |
| 71 | Holmberg et al., 2019 Sweden | Elucidate meanings of receiving assisted body care, as narrated by older persons living in a nursing home | Personal care | - N=12, older people participated (Mean age 91 years, age range 82-98 years, 83% female) - Facility-based care | Lifeworld | Older people described and experienced receiving assisted body care as an exposed and vulnerable position. Established nurse-patient relationship is a prerequisite, and if present could counter vulnerability in intimate personal hygiene situations |
| 84 | Housley et al., 2008 UK | Assess foot problems associated with diabetes among residents in care homes and develop appropriate foot care provision | Personal care | - N=90, residents (Mean age 84.2 years, 58% female) - Facility-based care | Prospective cohort | 95% of older people with diabetes had foot (i.e., skin and nails) pathology. 64% had ill-fitted footwear. Daily inspection of feet should be encouraged. Results suggested regular assessments in multidisciplinary teams to assess foot health as well measures for increased competence among nursing staff |
| 90 | Hunter & Levett-Jones, 2010 Australia | Present findings from a mixed method study which explored contemporary nursing practice in long term aged care in Australia | Fundamental nursing in general | - N=80, RNs (n=48) and nurse mangers (n=16), 14% had bachelor’s degree, 2% had master’s degree - Facility-based care | Mixed-methods | Results suggested fundamental nursing in facility-based care demand specialised skills and knowledge. Residents´ needs and activities were more clinically oriented. Participants highlighted the importance of including relatives. It was described as challenging to deliver person-centred care and integrate evidence into practice |
| 97 | King et al., 2020 Canada | Identify elements of assisting a frail senior to bath in a typical home bathroom, and assess risk of musculoskeletal injury and different techniques used | Personal care | - N=8, personal support workers (100% female) - Home-based care | Mixed-methods | Assisting older persons in personal hygiene is seen as a demanding activity, where 43% of the time is used in non-neutral postures and severe flexion and high back-loads. Further development of tools and techniques is needed to improve caregiver safety |
| 72 | Kuo et al., 2019 Taiwan | Explore nurses´ perceptions regarding providing psychological health care for older residents in long term care facilities | Psychosocial | - N=21, nurses working in long term care facilities (Mean age 38.4 years), 74,4% had a bachelor’s degree, average working experience as nurse was 8.7 years, standard deviation 6.45 years - Facility-based care | Qualitative descriptive | Results suggested that there is inadequate knowledge and skills for identifying older persons´ psychological health problems and provide optimal care. Nurses describe that they do not feel equipped to deal with psychosocial care. Educational strategies and training were suggested strategies to improve |
| 73 | Lindqvist et al., 2013 Sweden | Explore different nursing homes for the elderly what professionals with different responsibilities may consider as being important aspects of well-functioning daily oral care | Oral care | - N=23, RNs (n=10) nursing assistants (n=13) (Mean age 52 years, age range 32 – 65 years, 96% female) - Facility-based care | Qualitative descriptive | To ensure fundamental nursing, participants described a need for well-defined support, resources, clear responsibility, and role distribution. Implementation of oral care was not clearly defined. Oral care was described as rushed and neglected, RNs task-shifted oral care to nursing assistants |

| **No** | **Author, year, and country** | **Aim** | **Focus of Fundamental Nursing** | **Target group and setting** | **Design** | **Vital findings** |
| --- | --- | --- | --- | --- | --- | --- |
| 91 | Ludlow et al., 2020 Australia | Investigate aged care resident’s prioritization of care | Patients´ fundamental nursing needs | - N=38, older persons (Mean age 87.7 years, 65% female) - Facility-based care | Mixed-methods | Older persons reported that maintaining a sense of spirituality and self, information sharing, family involvement, being self-reliant, having timely access to support and management of conditions as important aspects of care |
| 85 | Martin et al., 2002 UK | Compare needs (met and unmet) and levels of dependency in residential care settings | Patients´ fundamental nursing needs | - N=75, older people (Mean age 80.6 years) - Facility-based care | Cross-sectional | Results indicated that the most common reported needs among older people are support in accommodation, household activities, nutrition, safety, personal finances, memory, and day-time activities. 25% (p<.05) had psychological needs |
| 74 | Mather & Bakas, 2002 USA | Examine nursing assistants’ perceptions about factors that either promote or inhibit continence care | Continence | - N=31, nurse assistants (100% female, age range 21 – 52 years) - Facility-based care | Qualitative descriptive | Participants highlighted the importance of established routines for optimal continence care (i.e., support for going to toilet every two hours), and that such care was based on cooperation and communication. Participants were concerned that excessive workloads, inconsistent co-operation, and lack of communication were barriers for optimal continence care |
| 75 | Mentes et al., 2006 USA | Explore perceptions of nursing home staff responsible for managing and monitoring oral intake in nursing home residents | Hydration | - N=28, certified nursing assistant (n=22), licensed vocational nurse (n=2), RN (n=2) allied healthcare personnel (n=2) (Mean age 35 years) - Facility-based care | Qualitative descriptive | Residents in risk of dehydration could be related to a variety of reasons, such as depression, loneliness, functional problems, and lack of support. Participants was concerned that loneliness were a major factor for reduced intake. Coordination and communication were reported as important strategies for optimal hydration care |
| 76 | Murphy et al., 2017 UK | Develop a model for understanding the complex nutritional problems associated with nutrition for people with dementia in care homes | Nutrition | - N=50, care staff (n=30), family carers (n=8), dietitians (n=3), speech and language therapists (n=3) - Facility-based care | Qualitative descriptive | Results suggested that a person-centred and collaborative approach to nutritional care could support the older persons nutritional needs. Nutritional care was seen as a complex and multi-dimensional fundamental need. Results indicated that availability, tools, resources, environment, involvement, consistency of care and sharing of information as important aspects of nutritional care |
| 95 | Næss et al., 2017 Norway | Describe the needs for nursing in a cohort of frail elderly persons and evaluate if provided nursing care adequately addressed their needs | Patients´ fundamental nursing needs | - N=83, older people (Mean age 87 years, 75% female) - Home-based care | Observational | Results indicate that 80% of older frail people live alone. More than 1/3 had severe limitations in fundamental needs (i.e., mobility, nutrition, and ADL). Results indicate that participants had complex health problems in many areas of fundamental nursing. Healthcare was perceived as too fragmented |

| **No** | **Author, year, and country** | **Aim** | **Focus of Fundamental Nursing** | **Target group and setting** | **Design** | **Vital findings** |
| --- | --- | --- | --- | --- | --- | --- |
| 86 | Orrell et al., 2008 UK | Describe residents´ perceptions of their own health and social needs compared with the views of the care home staff and the family carers | Patients´ fundamental nursing needs | - N=468, older people (n=387, mean age 85 years) and family caregivers (n=81) - Facility-based care | Cross-sectional | Results indicated that relatives reported more unmet care needs than the nursing staff did. Older people could often provide information about their needs. Nursing staff needed training in recognizing psychological distress, and situational awareness of older peoples´ relational and informational needs |
| 77 | Pasman et al., 2003 The Netherlands | Explore how nurses deal with inadequate food intake and aversive behaviour by patients and how nurses interacted with feeding problems | Nutrition | - N=94, residents with severe dementia - Facility-based care | Participant observation | Nurses feel responsible when patients stop to eat or eats too little. Nurses developed idiosyncratic approaches to the individual resident to optimise nutritional care. Results suggested that patients displaying aversive behaviour should be involved in a multidisciplinary approach, also including relatives |
| 99 | Persenius et al., 2008 Sweden | Study nurses’ perceptions of patient nutritional status assessment, screening tools and documentation in relation to nutrition | Nutrition | - N=131, RNs (Mean age 44.8 years) - Both home- and facility-based care | Multi-method | RNs assessed patients’ nutritional needs and used screening tools. The use of screening tools was not implemented in a consistent manner, consequently, not all older persons received assessments of their nutritional- needs or status. There was a clear need for increased knowledge |
| 87 | Reynolds et al., 2002 USA | Describe the palliative care needs of terminal home residents during the last 3 months of life | Patients´ fundamental nursing needs | - N=80, older people (Mean age 82 years, 61% female) - Facility-based care | Cross-sectional | Older persons receiving palliative care had complex fundamental nursing needs, such as personal care and continence care. Pain and fatigue were common physical symptoms. Psychological needs were seen in relation to depression and anxiety, where 30% reported unmet needs. Dying at home was preferred |
| 69 | Sjögren Forss et al., 2018 Sweden | Illuminate the experience of participation in nutritional care from the perspective of older people residing in nursing homes and RNs | Nutrition | - N=12, n=4 older people (Mean age 85.7 years, 75% female), RNs (n=8, mean age 44.1 years, 75% female) - Facility-based care | Qualitative descriptive | RNs emphasised the importance of supplying older persons with information in relation to nutrition. RNs described task-shifting of nutritional care to nursing assistants. Results encourage person-centred models of care to optimise nutritional care |
| 100 | Simmons & Patel, 2006 USA | Describe nursing home staff delivery of oral liquid nutritional supplements | Nutrition | - N=132, older people (Mean age 85.6 years, 82% female) - Facility-based care | Ethnography | Findings suggested that older persons did not receive the care they needed in relation to liquid oral supplements to sustain nutritional needs. Nursing staff used little time to encourage consumption (average 6 min. per meal) while the actual need is estimated to be 30 minutes. Increased involvement of more staff in care delivery was warranted |

| **No** | **Author, year, and country** | **Aim** | **Focus of Fundamental Nursing** | **Target group and setting** | **Design** | **Vital findings** |
| --- | --- | --- | --- | --- | --- | --- |
| 96 | Soini et al., 2006 Finland | Describe the nutritional status of Finnish home care patients, their problems related to eating, digestion, and diet and use of meals on wheels services | Nutrition | - N=178, older people (Mean age 83.5 years, 78% female) - Home-based care | Observational | 48% of older people receiving home-based care were at risk for malnutrition, while 3% were malnourished. 49% were found to be well nourished. Results indicated that home care patients had difficulty maintaining proper nutritional status. Comprehensive evaluation and assessments by nurses should be implemented |
| 88 | Suominen et al., 2005 Finland | Assess the nutritional status of all aged residents living in nursing homes in Helsinki by using the Mini Nutritional Assessment (MNA) | Nutrition | - N=2114 older people (Mean age 84.1 years, 80.2% female) - Facility-based care | Cross-sectional | 29% of older people in facility-based care suffered from malnutrition, and 60% were at risk for malnutrition. Dementia, pressure ulcers or stroke were associated with impaired nutritional status. Few of the older persons in the sample received energy-dense food and option of nutritional supplements |
| 78 | Taunton et al., 2005 USA | Describe the care provided to nursing home residents with urinary incontinence | Continence | - N=105, older people (n=17, age range 67 - 99 years), next of kin (n=16) and healthcare professionals (n=72) - Facility-based care | Qualitative emergent case | Despite two-hour toileting being policy, none of the residents were assisted to the toilet on a two-hour basis. Nurses had few alternatives to two-hour schedule and continence pads. Most participants in the sample did not have training beyond basic skills. Lack of resources were associated with poor person-centred care |
| 79 | Taylor et al., 2014a Australia | Explore factors amongst residents that influence mobility, and explore how those factors may inform staff training developments in resident-centred mobility care | Mobility | - N=15 older people (Mean age 86 years, age range 61-96, 73% female) - Facility-based care | Ethnography | Results indicate that residents’ value mobility and had extensive needs for support and safety. Poor communication and negotiation could lead nurses to assuming control over residents’ mobility. Results suggested that nurses should implement a model of care based on residents’ attitudes and autonomy |
| 80 | Taylor et al., 2014b Australia | Explore factors influencing the quality of mobility care by integrating factors related to key aspects of safety, mobility optimization, and relationship | Mobility | - N=43, n=15 older people (Mean age 86 years, age range 61-96, 73% female), and healthcare professionals (n=28) - Facility-based care | Ethnography | Findings suggested that person-centred models of care may improve the quality of mobility care and associated outcomes. Risk assessments, staff familiarity, staff stability and teamwork could provide an enriched environment for quality mobility care |
| 89 | Van der Ploeg et al., 2013 The Netherlands | Describe the difference in number and type of care needs of people with and without dementia living in residential care | Patients´ fundamental nursing needs | - N=187, older people, (Mean age 86.6 years, 73.2% female) - Facility-based care | Cross-sectional | Results highlighted that older persons with dementia had more comprehensive care needs than those without dementia. Results indicated that common needs for both groups were accommodation, household activities and food. Needs among persons with dementia was linked to activity (p<.001), company (p<.001), continence care (p<.01) and inadvertent-self harm (p<.01) |

| **No** | **Author, year, and country** | **Aim** | **Focus of Fundamental Nursing** | **Target group and setting** | **Design** | **Vital findings** |
| --- | --- | --- | --- | --- | --- | --- |
| 81 | Wu et al., 2012 Taiwan | Explore the nurses’ attitudes towards physical activity care for older people in long term aged facility care | Mobility | - N=20 nurses (Mean age 40.5 years, 100% female) - Facility-based care | Qualitative explorative | Physical activity and mobility care were seen as beneficial for residents’ overall health, and could be linked to physical, psychosocial, and relational outcomes. Results suggested that positive activities, such as singing, gardening, and playing games was efficient in engaging and involving residents to be physical and stimulated |
| 82 | Ødbehr et al., 2015 Norway | Investigate how nurses and care workers provide spiritual care for people with dementia in nursing homes | Psychosocial and relational | - N=31, RNs (n=16), care workers (n=15)   Facility-based care | Qualitative explorative | Participants stated that the physical touch was an aspect of spiritual care and could be revolved around activities such as sitting at the bedside, holding hands, or a friendly smile during nursing activities. Less attention was paid to religious care, which was seen as something different than spiritual care |
