# Supplementary material for: What about the fundamentals of nursing—its interventions and its continuity among older people in need of home- or facility-based care: a scoping review
Source: BMC Nurs. 2024 Jan 22;23:59. doi: 10.1186/s12912-023-01675-1 (PMC10801980; doi:10.1186/s12912-023-01675-1)
Supplement: Supplementary file 4 — Additional file 4. Overview of papers answering question three. [file 12912_2023_1675_MOESM4_ESM.docx]

| **No** | **Author, year, and country** | **Aim** | **Focus of Fundamental Nursing** | **Target group and setting** | **Design** | **Intervention components**  **Models/Pathways/Guidelines and Outcome** | **Vital findings** |
| --- | --- | --- | --- | --- | --- | --- | --- |
| 102 | Christensson et al., 2003,  Sweden | To test implementation of educational nutritional programme would lead to change in nursing staff attitude | Nutrition | - N=368, first occasion n=176 (RNs n=7, nurse aides n=169). Second occasion: n=192 (RNs n=8, nurse assistants n=184) - Facility-based care | Experiment with intervention group and control group without randomization | Experiment group received an educational programme in how to identify individual needs, how to use a dietary plan and a food record. RNs received training on how to use the Mini Nutritional Assessment (MNA) form, calculation of energy requirements, underlying causes of nutritional problems, interfering medication and how to structure nutritional documentation. Results in comparing control and experiment group showed minimal effect. The highest change was measured during initial phase of intervention | Both experiment and control group filled out the “Staff Attitudes to Nutritional Nursing Care” scale, in which results indicate that nursing staff in general had positive attitudes towards nutritional care. Social influence, culture and group norms are likely main factors influencing nutritional care |
| 105 | Frändin et al., 2015  Sweden, Norway, Denmark | Evaluate long-term effects on activities of daily living, balance function, physical activity, performance, falls related self-efficacy, well-being and cognitive function after a individually tailored restorative intervention | Mobility, personal care, and ability | - N=322, older people (Mean age 85 years, 89% female) - Facility-based care | Multicentre randomized controlled clinical trial with parallel-group design in Scandinavia | Experiment group received tailored physical and daily activities based on individual treatment goals. The most common intervention was focusing on limitations in mobility and personal care. Three months after the intervention period ended, experiment group dropped back to almost baseline | Intervention supported by occupational and physical therapists. Results indicate that continuous physical exercise adjusted to functional level and individual needs is crucial for maintaining functional ability among older persons in facility-based care |
| 101 | Ibrahim et al., 2019,  Australia | Test a conceptual model targeting older persons overall needs in facility-based care | Personal-, social- and patient involvement | - N=382, nursing staff (n=77) and older people (n=51), relatives (n=29) and other allied healthcare professionals (n= 225) - Facility-based care | Feasibility testing/assessment of model through stakeholder involvement | Model provided practical ways to operationalise the different aspects of the care needed by residents while incorporating the complexity faced by service providers to avoid shortcomings in fundamental nursing. The conceptual model components in relation to physical needs and dementia management are transferrable to other LTC contexts, social inclusion and legal rights are entangled in constitution and culture in each country. The study does not measure outcome | The model encompassed a comprehensive and person-centred approach to care, including physical, social needs, function, and quality of life, additionally including a legal and human rights perspective. The model show promise in delivering a person-centred care to older people with complex health issues, enabling increased quality of life and thriving in facility-based care |
| 104 | Lannering et al., 2017,  Sweden | Describe nursing staff experiences of preventive work by using a structured preventive care process as outlined by intervention “Senior Alert” | Prevention of falls, malnutrition, and pressure ulcers | - N=44, RNs (n=26) assistant nurses, n=17) and occupational therapists (n=1) - Both home- and facility-based care | Qualitative descriptive design, data gathered through focus groups (n=8) | The “Senior Alert” (SA) preventive intervention supports nursing staff to assess risk for falls, malnutrition, and pressure ulcers and to implement team-based interventions and follow-up results. The “Senior Alert” uses validated assessment instruments, e.g., Mini Nutritional Assessment Scale, Modified Norton Scale and Downton fall risk index. If patient is at risk, underlying causes should be analysed, and a team-based approach implement interventions based on evidence-based factors and frequent evaluate interventions | Preventive work among frail older people in LTC is complex. Assessment scales does not reflect reality as the older population in LTC have changed as opposed to the older population the scales initially were developed towards. Nursing assistants in facility-based LTC experienced that there were too many tasks to fully utilize the “Senior Alert” intervention. Nurses in home-based care highlighted the needs for increased support in fundamental nursing interventions to prevent malnutrition |

| **No** | **Author, year, and country** | **Aim** | **Focus of Fundamental Nursing** | **Setting and Participants** | **Design** | **Intervention components**  **Models/Pathways/Guidelines and Outcome** | **Vital findings** |
| --- | --- | --- | --- | --- | --- | --- | --- |
| 106 | Mentes & Culp, 2003, USA | Test whether the use of a hydration management intervention meets older persons hydrational needs in facility-based care, and if acute confusion and urinary tract infections was reduced | Hydration | - N=49, older people (Mean age 81,8 years, 55% female) - Facility-based care | Quasi-experiment with experiment and control group without randomization. 8-week intervention period | The hydrational intervention is based on individual calculation of daily fluid goal adjusted for weight, in additional to an extensive baseline assessment and strategies for ensuring that fluid goals were met, such as standardized fluid intake (180ml) with medication, fluid rounds twice a day, and “happy hour” or “tea-time” twice a week in the late afternoon. Weekly urinalyses and 24-hour fluid record were used to determine if fluid intake was adequate. Experiment group had fewer hydration linked events as compared to control group | Maintaining adequate hydration among older persons in nursing homes required consistent fundamental nursing and persistent point-of-care nurses. Hydrational care can be neglected with negative outcomes for the patient (dehydration, infection, constipation, and confusion). Hydration requirement should be tailored to each individual patient. Residents need be offered fluids throughout the day |
| 110 | Parsons et al., 2013,  New Zealand | Determine whether a restorative intervention would result in increased physical ability and social support through individually tailored activity and support among older people receiving home-based care | Mobility and personal care | - N=205, older people (Mean age 79.1 years, 60,8% female) - Home-based care | Experiment with randomized control and intervention group | Patients went through a support needs assessment where individual needs were identified, and goals were set using the Towards Achieving Realistic Goals in Elders Tool (TARGET). Areas of deficit were targeted, such as falls risk, muscle atrophy, and difficulties with personal care. Goals were set within the dimension’s mobility, self-care, pain/discomfort and anxiety/depression. Goals were set in both a short- and long-term perspective in a goal ladder by the need’s assessor in co-operation with the patient, this information was passed down to the home-based care organization created concrete instructions for the home care aides | Participants in the experiment group had significant increase in physical function and gait speed as compared to control group. Results indicate that home-based care can be re-oriented towards models of care focusing increased independence and identifying factors needed to improve patient outcomes |
| 107 | Schnelle et al., 2002  USA | Assess the functional benefits of participating in a Functional Incidental Training (FIT) intervention for 8 months, and how older persons participating responded. A secondary aim was to establish how much time was needed to implement the intervention and translate the intervention into nursing home practice | Mobility, hydration, and continence care | - N=190, older people (Mean age 87.5 years, 83.5% female) - Facility-based care | Experiment with randomized control and intervention group | The FIT intervention was delivered 2-hours daily, 5 days a week, in a total of four care episodes per day. For each care episode, residents were prompted to the toilet and changed in needed. Before or after continence care, patients were encouraged to walk, or wheelchair supported sit-to-stand exercises. Once daily, upper body resistance training was provided. Before and after each care episode, fluids were offered. Outcomes were assessed in relation to incontinence, endurance, strengths, and labour requirements needed for intervention implementation | The majority of intervention participants experienced improved mobility, upper body strength and continence. The staffing requirements to implement the intervention exceeds available in most facility-based care. Inadequate staffing levels impedes successful transfer of the FIT intervention and other evidence based and effective interventions in facility-based care |
| 108 | Simmons & Ouslander, 2005  North America | Evaluate the satisfaction of an intervention targeting mobility and continence care among older persons in nursing homes and their relatives | Mobility and continence care | - N=97, older people (Mean age 89.2 years, 89% female), relatives (n=97) - Facility-based care | Experiment with randomized control and intervention group combined with interviews and observations | Participants underwent a baseline assessment focusing mobility and continence and experiment group underwent an 8-week intervention period where intervention staff provided the intervention 2 hours during dayshifts 5 days a week, divided into four care episodes per day. In each care episode, patients prompted to the toilet and changed if necessary, and then supported to walk and repeated sit-to-stand exercises | Results indicated that both patients and relatives report a preference for care frequency higher than that provided by nursing staff. To improve quality of mobility and continence care, involvement of patients in fundamental nursing is pivotal |

| **No** | **Author, year, and country** | **Aim** | **Focus of Fundamental Nursing** | **Setting and Participants** | **Design** | **Intervention components**  **Models/Pathways/Guidelines and Outcome** | **Vital findings** |
| --- | --- | --- | --- | --- | --- | --- | --- |
| 109 | Stolt et al., 2011  Finland | Investigate the knowledge of nursing staff regarding foot care, their foot care activities, and the health of residents in a nursing home before and after and educational intervention | Personal care | - N=59, older people (n=43, Mean age 86.4 years, 84% female) and nursing staff (n=16) - Facility-based care | Experiment with intervention but without control group or randomization supported with individual interviews and pre-test post-test observations | The intervention was comprised of lectures, demonstrations and questions designed to improve knowledge of foot care, assessment of footwear, diabetic footcare and skin and nail care. Intervention was divided between two 90-minutes sessions with distinct topics. Outcomes were measured using the Foot Health and Footwear Structured Assessment Form and the Nursing Staff Knowledge and Foot-Care Activities questionnaire | Results indicate that nursing staff partially increased knowledge, mainly seen in skin health, the amount of hammer toes and dry skin decreased, and three out of four residents needed podiatric services. The educational intervention revealed a need for optimization, but demonstrated that nursing staff knowledge can be changed, and residents foot health can be increased |
| 103 | Törmä et al., 2017  Sweden | Compare two different implementation strategies of external facilitation versus educational outreach visits when introducing nutritional guidelines in nursing homes | Nutrition | - N=105, nursing aides and nurse assistants (Mean age 43.2 years) - Facility-based care | Experiment with intervention groups but without control group or randomization supported with pre-test post-test observations | Outcomes were assessed through the Five Aspects Meal Model (FAMM) comprised of five distinct topics related to the mealtime experience. The intervention was delivered by educational outreach visit (EOV) groups and external facilitation groups (EF). The EOV group received a 3-hour lecture about nutritional guidelines, clinical outcome measures and plan for introducing the guidelines. The EF group met with a facilitator every 3-4 weeks over the course of a year. The EF strategy consisted of feedback on baseline data, practice audit and feedback on mealtime observations and dietary assessments. | The EF group improved mealtime ambience (laying the table), offering a choice of beverage, offering more fluids and the quality of serving of the meal. Improvements were detected in interaction between staff, less noise from kitchen in which the EF group was favourable to the EOV group |
